# Identification of highly synchronized subnetworks from gene expression data

**DOI:** 10.1186/1471-2105-14-S9-S5

**Published:** 2013-06-28

**Authors:** Shouguo Gao, Xujing Wang

**Affiliations:** 1 Department of Physics, University of Alabama at Birmingham, Birmingham, AL, 35294, USA; 2 The Comprehensive Diabetes Center, University of Alabama at Birmingham, Birmingham, AL, 35294, USA

## Abstract

**Background:**

There has been a growing interest in identifying context-specific active protein-protein interaction (PPI) subnetworks through integration of PPI and time course gene expression data. However the interaction dynamics during the biological process under study has not been sufficiently considered previously.

**Methods:**

Here we propose a topology-phase locking (TopoPL) based scoring metric for identifying active PPI subnetworks from time series expression data. First the temporal coordination in gene expression changes is evaluated through phase locking analysis; The results are subsequently integrated with PPI to define an activity score for each PPI subnetwork, based on individual member expression, as well topological characteristics of the PPI network and of the expression temporal coordination network; Lastly, the subnetworks with the top scores in the whole PPI network are identified through simulated annealing search.

**Results:**

Application of TopoPL to simulated data and to the yeast cell cycle data showed that it can more sensitively identify biologically meaningful subnetworks than the method that only utilizes the static PPI topology, or the additive scoring method. Using TopoPL we identified a core subnetwork with 49 genes important to yeast cell cycle. Interestingly, this core contains a protein complex known to be related to arrangement of ribosome subunits that exhibit extremely high gene expression synchronization.

**Conclusions:**

Inclusion of interaction dynamics is important to the identification of relevant gene networks.

## Background

Life is a transient dynamic phenomenon. Biological functions and phenotypic traits, including disease traits, stem from the interactions across multiple scales in the living system. Therefore characterizing the condition-dependent interactions and emergent dynamics are important in the identification of relevant elements to a given biological process.

Recently, a number of computational methods have been developed to identify the condition specific protein-protein interaction (PPI) subnetworks, through integration of generic PPI data (typically obtained from an interactome database) and condition-specific gene expression data [1]. For instance, by integrating yeast PPI networks with gene expression data, Han et al. showed that some modules are active only at specific times and locations [2]. Qi et al. suggested that such approach enables the identification of subnetworks that are active under certain conditions [3]. In a cell cycle study by de Lichtenberg et al, it was found that the cell cycle-regulated and constitutively expressed proteins form protein complexes at particular time points during the cell cycle [4]. In these studies correlation in expression or similar measures are usually used to capture the condition specific gene interaction [3-9]. More recently, a number of studies focused on integration of PPI networks with time course expression data to identify subnetworks that exhibit meaningful dynamic changes in transcription. In a study of yeast metabolic oscillation by Tang et al [5], the active PPI network is first constructed for each time point (out of a total of 36 time points) through identification of interacting protein pairs whose corresponding genes exhibit a certain significant pattern in expression at that time point. Then Markov clustering algorithm is applied to create candidate functional module of each network. These modules were found to have much more significant biological meaning than those derived using static PPI networks only [5]. In another study, Jin et al [6] defined a dynamic network module to be a set of proteins satisfying two conditions: (1) they form a connected component in the PPI network; and (2) their expression profiles exhibited time-shifted and local similarity patterns as evaluated using an time-warping dynamic programming algorithm. Using yeast as a model system and time course expression data from multiple experiments, they then showed that the majority of the identified dynamic modules are functionally homogeneous, and many of them shed light on the sequential ordering of the molecular events in the cellular system of yeast [6].

Understanding cellular physiology from a dynamic and systems perspective is obviously very important and valuable as demonstrated by these studies and many others [10]. Incorporating time course data is a necessity along this direction. They not only capture how a whole system evolves over time, but also contain rich information regarding the coordination, namely, interaction, of the different elements in the system. The measurements from different time points are not independent of each other; this is in contrast to static measurements of different samples, or of the same sample under different conditions. However, most of the existing studies either construct active networks independently at each time point [5], or rely on pattern similarity measures to infer interaction which ignores the inter-time point dependence [6]. Overlooking the interdependence among the time points not only loses sensitivity toward detecting relevant interactions but could also lead to erroneous predictions [11,12].

In this study we investigate the application of an idea rooted in statistical physics and non-linear dynamics to characterize the state of gene interaction networks and use it to identify relevant subnetworks. We regard active subnetworks to be those showing high degree of differential expression, and high synchrony in expression changes (i.e., coordination in the timing of expression changes) among the members. The phase locking analysis will be utilized to evaluate expression synchrony, and to capture the dynamic interaction structure. Recently we found that the phase locking metric can identify interacting gene pairs more efficiently than correlation [11].

Previously, we proposed a Pathway Connectivity Index (PCI) to represent the activity of pre-defined pathways, such as those defined in KEGG and Biocarta. PCI utilizes expression information of all genes in a pathway, as well as the topological properties of its interaction networks. Its advantages have been demonstrated [13]. This metric was later implemented in a software tool entitled Topological Analysis of Pathway-Phenotype Association (TAPPA). Here to capture contributions from topological characteristics of the dynamic interaction network, we integrate the phase locking analysis into PCI to define a novel metric: the Topology-Phase Locking (TopoPL) analysis [13]. With both simulated and real yeast expression data during cell cycle, we will demonstrate the merits of TopoPL.

## Methods

### Simulation study

Simulation utilized the sample expression data gal80R given in Cytoscape (http://cytoscape.org/). There are 331 genes and 361 interactions in this network. Within it, we randomly selected subnetworks at three different sizes n (n = 40, 60, 80), as condition-responsive. In each responsive subnetwork m% (80%, 90%, 100%) of genes are defined to be active. The significance values of active genes were assigned randomly with top n×m% significance values in gal80R, and that of the other genes were randomly sampled from the rest of the significance values. The phase locking index λ (see 2.3) of the interactions in the predefined responsive subnetwork were sampled from $N\left(0.8,0.5\right)$, i.e. a normal distribution with μ = 0.8, σ = 0.5; while λ for the remaining edges were sampled from N(0.4,0.3). The choice of these values was based on the distribution of the λ values of gene pairs in protein complexes and of randomly selected gene pairs. For protein complexes we used the MIPS annotation (http://mips.helmholtz-muenchen.de/genre/proj/yeast) edited by Gerstein Lab (http://www.gersteinlab.org/proj/bottleneck/mips.txt).

A gene of the predefined responsive subnetworks that is in the TopoPL-identified subnetwork is considered a successful identification. This procedure was repeated 10 times and the true positive (TP, sensitivity) rate was defined to be the number of successful identifications divided by the size of the predefined network n. The false positive (FP, specificity or precision) rate was estimated as the number of false identifications divided by the size of the identified subnetwork. The F score is a measure of a test's accuracy. It considers both the precision and the sensitivity of the test:

$$F=\frac{specificity\text{*}sensitivity}{\left(specificity+sensitivity\right)/2}$$

We used the average sensitivity, specificity and F score to measure the performance of TopoPL. The performance is also evaluated with Receiver Operating Characteristic (ROC) curve, a plot of the true positive rate against the false positive rate [11].

### Gene expression and protein-protein interaction data

Gene expression data was downloaded from EMBL's Huber group (http://www.ebi.ac.uk/huber-srv/scercycle/). It is a time course study of yeast cell cycle, where cells were arrested using alpha factor or cdc28. The alpha factor dataset contains 41 time points and the cdc28 dataset contains 44 time points, both at 5-minute resolution. These datasets provide strand-specific profiles of temporal expression during the mitotic cell cycle of S. cerevisiae, monitored for more than three complete cell divisions [14]. Yeast PPI data were downloaded from BioGRID (thebiogrid.org, version 3.1.69).

### Phase locking analysis

The details of definitions and steps of the phase locking analysis was described in our previous work [11] and briefly summarized here. Given a time series *s*(*t*), its Hilbert transformation is given by

(1) $$s_{H}\left(t\right)=\frac{1}{\pi }\text{PV}\int_{-\infty }^{\infty }\frac{s\left(t\right)}{t-\tau }d\tau$$

where PV stand for Cauchy Principal Value of integration. The corresponding analytical signal can then be constructed by:

(2) $$s\left(t\right)+is_{H}\left(t\right)=A\left(t\right)e^{i\varphi \left(t\right)}$$

where the instantaneous phase $\varphi \left(t\right)$ is thus uniquely determined. For two time series with instantaneous phase $\varphi_{i}\left(t\right)$ and $\varphi_{j}\left(t\right)$, their cyclic relative phase is determined by

(3) $$\Psi \left(t\right)=\left(\varphi_{i}\left(t\right)-\varphi_{j}\left(t\right)\right)\text{mod}\left(2\pi \right)$$

If two time series interact with each other, there will be rhythmic adjustment resulting in phase locking: $\Psi =\Psi_{0}$ is a constant. To evaluate the significance of phase locking, we utilize the circular mean of the phase difference

(4) $$\lambda =\left|\text{exp}\left(i\Psi \left(t\right)\right)\right|=\left|\left(\left(\frac{1}{t_{N}}\right)\overset{N}{\underset{l=1}{\sum }}\text{exp}\left(i\Psi \left(t_{l}\right)\right)\right)\right|$$

In a perfect locking $\lambda =\left|\text{exp}\left(i\Psi_{0}\right)\right|=1$, and λ→0 when $\Psi \left(t\right)$ is randomly distributed. *λ *offers a new measure to infer potential interaction between gene pairs [11].

### TopoPL

For each gene *i*, the EDGE software [15] was used to calculate $p_{i}$, the significance of its expression changes during the time course study. We convert $p_{i}$ to a z-score through $z_{i}=\emptyset^{-1}\left(1-p_{i}\right)$, where $\emptyset^{-1}$ is the inverse normal CDF. Let $A^{\left(P\right)}=\left({a^{\left(p\right)}}_{ij}\right)$ be the adjacency matrix of genes in a PPI subnetwork and $A=\left(a_{ij}\right)=\left({a^{\left(p\right)}}_{ij}*\lambda_{ij}\right)$, TopoPL defines the overall activity of a subnetwork with:

(5) $$z_{A}^{TopoPL}=\sum_{i\in A}\sum_{j\in A}|z_{i}{|}^{0.5}*a_{ij}*|z_{j}{|}^{0.5}*sgn\left(z_{i}+z_{j}\right)$$

$z_{A}^{TopoPL}$ captures the dynamic topological property of the subnetwork, and hub genes (genes with high network degree) contribute more to this metric. $|z_{i}{|}^{0.5}*a_{ij}*|z_{j}{|}^{0.5}*sgn\left(z_{i}+z_{j}\right),i\neq j$ can be regarded as the "activity measurement" of the interaction. Gene pairs with significant and synchronized expression changes, and whose gene products interact, contribute more to the activity of the subnetwork.

This metric is an improved version over the PCI that we previously proposed to identify active pathways from gene expression data [13]: $PCI=\sum_{i,j}|x_{is}{|}^{0.5}*a_{ij}*|x_{js}{|}^{0.5}*sgn\left(x_{is}+x_{js}\right)$, where $x_{is}$ is normalized log expression measurement of gene *i *in sample *s*, and $\left(a_{ij}\right)$ is the adjacency matrix of the PPI network of genes in the pathway. The merit of PCI has been demonstrated in previous works [13]. To reduce the potential impact on the network measure from residual inter-sample and inter-array biases after normalization, here we adopted the non-parametric measure $z_{i}$ in place of $x_{is}$. A similar metric to Eq. (5) was developed recently by us to predict candidate disease genes for type 1 diabetes, where $z_{i}$ is the z-score of disease relevance of gene *i*. There again we demonstrated the advantage of incorporating network structural information [16].

Obviously, $z_{A}^{TopoPL}$ increase with the number of nodes and edges. To adjust for network size and density, we use the following equation

(6) $$z_{A}^{TopoPL}\to z_{A}^{TopoPL}*\frac{1}{\left(\#nodes+\#edges\right)}$$

We implemented the searching procedure based on simulated annealing. The pseudocode of the algorithm is described below:

**Input: **the entire network $G_{0}=\left(V,E\right)$; a set of parameters for running simulated annealing: start temperature $T_{start}$ (= 1 in this study), end temperature $T_{end}$ (= 1e-8 in this study), number of iterations  N.

**Output: **the subnetwork with the highest score.

**Steps**: initialize each node with its expression significance score $z_{i}$ and each edge with its phase locking index; select the largest connected component (subnetwork) $G_{out}$ from top 10% significant nodes of $G_{0}$; calculate score of $G_{out}$ and obtain its score $z_{out}^{TopoPL}$; then run the following:

For i = 1 to N, Do

Calculate the current temperature $T_{i}=T_{i}*0.8^{1/N}$; $G_{try}\leftarrow G_{out^{\prime }}$

Exit loop if $T_{i}<T_{end}$

Randomly pick a node n∈V

IF ($n\in G_{try}$), remove n from $G_{try}$;

ELSE add n to $G_{try}$;

Calculate score $z_{try}^{TopoPL}$for the largest connected component of $G_{try}$;

Calculate $\Delta =z_{try}^{TopoPL}-z_{out}^{TopoPL}$;

IF Δ> 0, then$G_{out}\leftarrow G_{try}$;

ELSE, accept $G_{out}\leftarrow G_{try}$ with the probability$p=e^{\Delta /T_{i}}$;

END

These steps can be iterated to identify subnetworks with the next highest scores and so on.

In this study we compared TopoPL with two other methods: (1) The commonly used network scoring method that sums significance levels of all genes in the network (hereafter referred to as the Additive scoring method):

(7) $$z_{Additive}=\sum_{i\in A}z_{i}$$

(2) A metric that we previously proposed in our TAPPA software package [13] (hereafter referred to as the TAPPA scoring method) that only utilize the topological characteristics of the PPI network:

(8) $$z_{Topo}=\sum_{i\in A}\sum_{j\in A}|z_{i}{|}^{0.5}*{a^{\left(p\right)}}_{ij}*|z_{j}{|}^{0.5}*sgn\left(z_{i}+z_{j}\right)$$

## Results

### Simulation study

**Figure 1:**
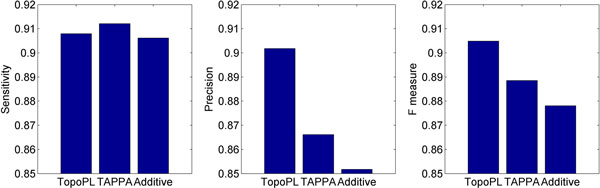
**Performance of TopoPL, TAPPA and Additive**. Three approaches have similar sensitivity, but TopoPL has higher precision. Results are from the simulated data.

**Figure 2:**
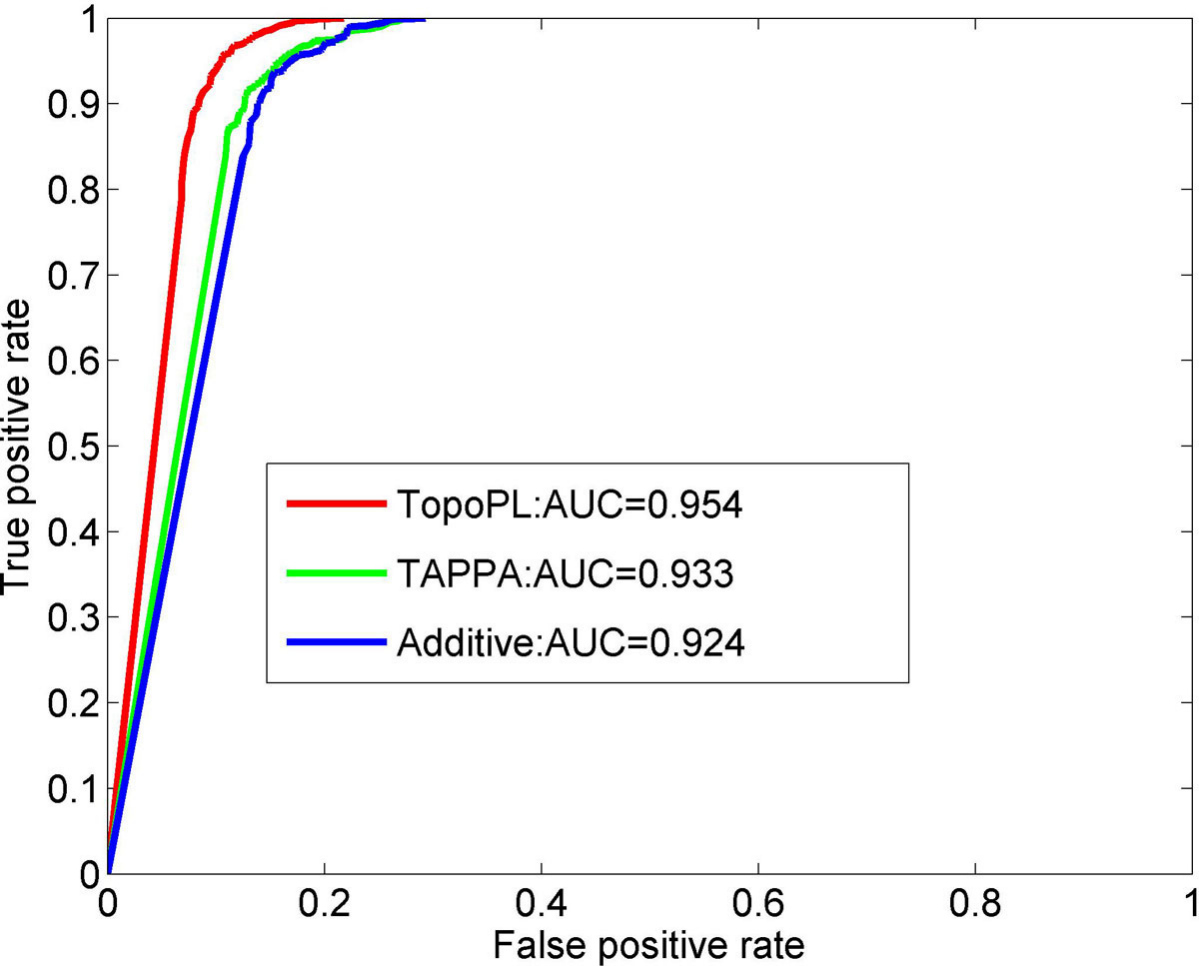
**ROC plot of TopoPL, TAPPA and Additive**. TopoPL has the highest AUC. Results are from the simulated data.

Using the simulated yeast gene expression data, we compared TopoPL with two other methods: (1) Additive scoring method (see definition Eq. (7) in Methods); and (2) TAPPA (see definition Eq. (8) in Methods) [13]. Additive does not use any structural information of the network, TAPPA uses only predefined static network structure ignoring the dynamic, condition-specific changes in interaction patterns. Figure 1 summarizes the average sensitivity, precision and F score from all simulated data: 10 replicates each of three network sizes (*n *= 40, 60, 80), at three states of activity (m = 80%, 90%, 100%). Though the three methods have similar sensitivity, the precision of TopoPL is higher. F scores showed that TopoPL performs better than TAPPA and Additive. The ROC curves also indicate that TopoPL performs better than the other two approaches, with the highest Area Under Curve (AUC), as shown in Figure 2.

### Yeast cell cycle data

After 100,000 iterations (N=100,000), TopoPL identified a subnetwork of 524 genes and 2078 edges with the alpha factor dataset (in the following sessions, unless specified otherwise, we only report results from the alpha factor dataset; the cdc28 dataset gives very similar results). We performed the GO term enrichment analysis with topGO package in Bioconductor (http://www.bioconductor.org) to investigate how well the identified subnetwork captured the relevant functional modules [17]. The most significant "Biological Process" GO terms are listed in Table 1. Many cell cycle, growth, and division-related processes were enriched in this subnetwork, such as GO:0042254 (ribosome biogenesis); GO:0007049 (cell cycle); GO:0022613 (ribonucleoprotein complex biogenesis); GO:0000278 (mitotic cell cycle); GO:0000280 (nuclear division). Almost all top terms are cell cycle related. Ribosomes are "factories" of protein synthesis, and synthesis of ribosomes is a key control point for the regulation of cell growth and division.

**Table 1:** Top 10 GO Biological Processes terms significantly enriched in the subnetwork identified during yeast cell cycle.

GO ID	GO name	P value
GO:0042254	ribosome biogenesis	1.04E-13

GO:0007049	cell cycle	9.31E-13

GO:0022613	ribonucleoprotein complex biogenesis	1.46E-12

GO:0000278	mitotic cell cycle	2.07E-11

GO:0000280	nuclear division	1.00E-08

GO:0022402	cell cycle process	2.81E-08

GO:0044085	cellular component biogenesis	3.00E-08

GO:0051301	cell division	3.65E-08

GO:0048285	organelle fission	5.13E-08

GO:0006364	rRNA processing	1.67E-07

P values were Bonferroni corrected.

Presently, there is no "gold standard" to evaluate the biological relevance of network modeling algorithms. Here we investigated the functional enrichment of the proteins in the identified subnetworks [9], and compared to that obtained using Additive and TAPPA. The p values (Bonferroni corrected) of the top 2 terms are 3.33E-13 and 6.5E-12 with TAPPA, and 3.05E-8 and 3.13E-8, with Additive, respectively. TAPPA's are slightly larger than TopoPL, but Additive gave much larger p values. This indicates that including interaction structure, especially its dynamics, improves the sensitivity at identifying biologically relevant gene subnetworks.

It has been demonstrated that hub genes and high betweenness genes (*i.e*. genes having high number of shortest paths passing through) play important roles in gene networks [18]. Table 2 listed the top 30 high-degree and high-betweenness nodes from the identified subnetwork. Though not been annotated with cell cycle, HEK2 is a RNA binding protein involved in asymmetric localization of the mRNA of ASH1, a transcription factor that acts to specify daughter cell fate in mating-type switching [19]. Dsn1 has been annotated with cell cycle, it is important for chromosome segregation in S. cerevisiae [20]. TPK1 has been annotated with the cell cycle GO terms. It is a cAMP dependent protein kinase which mediates basic cellular processes, such as the yeast-to-hypha transition and cell cycle regulation [21]. NOP15 is also annotated with cell cycle GO terms. The transcription level of NOP15 is an important determinant of the productivity of RNA and its increased transcription provides an effective approach to obtain higher RNA yields in yeast [22].

**Table 2:** Top 30 genes with highest degrees or betweenness in the identified subnetwork.

Degree			Betweenness		
**Official Symbol**	**degree**	**Cell cycle?**	**Official Symbol**	**betweenness**	**Cell cycle?**

HEK2	155		HEK2	61898	

DSN1	76	YES	DSN1	24078	YES

NOP15	70	YES	TPK1	12196	YES

CIC1	60		HSP82	10612	

NOP7	58	YES	YPL141C	5798	

RRP5	54		ORC1	5767	

NOC2	54		RRP5	5641	

ERB1	52		KSS1	5560	YES

RPF2	52		RAD53	4074	YES

BRX1	52		DBF2	3755	YES

NUG1	51		CLB2	3698	YES

TPK1	50	YES	CDC5	3218	YES

HAS1	50		NOP15	2904	YES

NOP2	50		HHF1	2902	

ORC1	49		BUD21	2745	

NSA1	49		SHE2	2650	

YTM1	46		SML1	2432	YES

RLP7	45		RRP1	2401	

RRP1	44		HHT1	2376	YES

MRT4	42		HAS1	2221	

HSP82	40		YGR130C	2207	

DRS1	38		MPP10	2116	

MAK21	38		SPO12	1941	YES

PUF6	36		CSM1	1842	YES

NOP4	36		RCK1	1808	YES

RAD53	34	YES	RFA1	1770	YES

RLP24	34		CDC20	1737	YES

EBP2	34		ACE2	1709	YES

RPF1	32		YAK1	1706	

MPP10	31		CLN2	1701	YES

**Figure 3:**
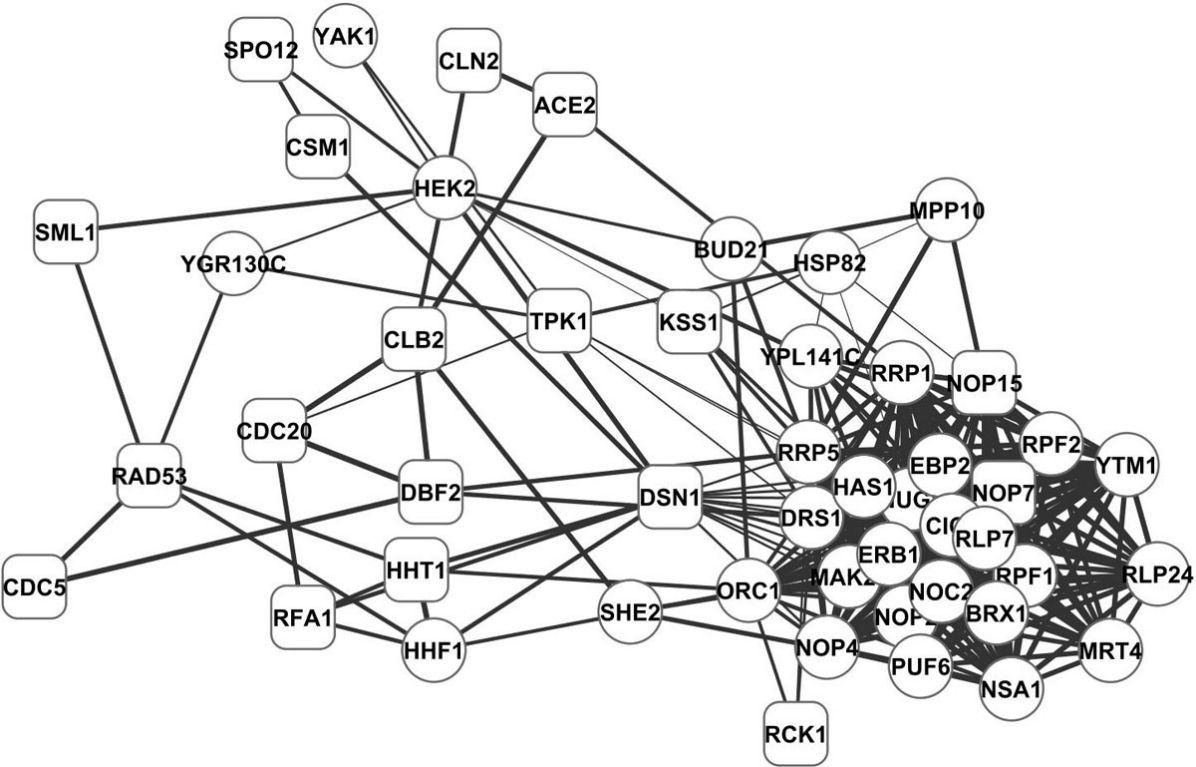
**Core of the identified subnetwork**. Rectangles denote cell cycle genes and thicker lines indicate higher synchronization.

The top 30 high-degree and high-betweenness nodes from the identified subnetwork and their interactions are presented in Figure 3. We hypothesize that they constitute a relevance core to yeast cell cycle, and provide a holistic picture of the primary molecular basis of cell cycle. In the core there are 18 genes annotated with GO:0007049 cell cycle (round rectangles), this rate (18 out of 39) is higher than that of the whole identified subnetwork (128 out of 524, a 1.9 fold enhancement, *p *= 0.11), and that of all genes in yeast (612 out of 5286, *p *= 0.00013). These results suggest that degree and betweenness can be utilized to further improve the performance of functional gene module identification.

**Figure 4:**
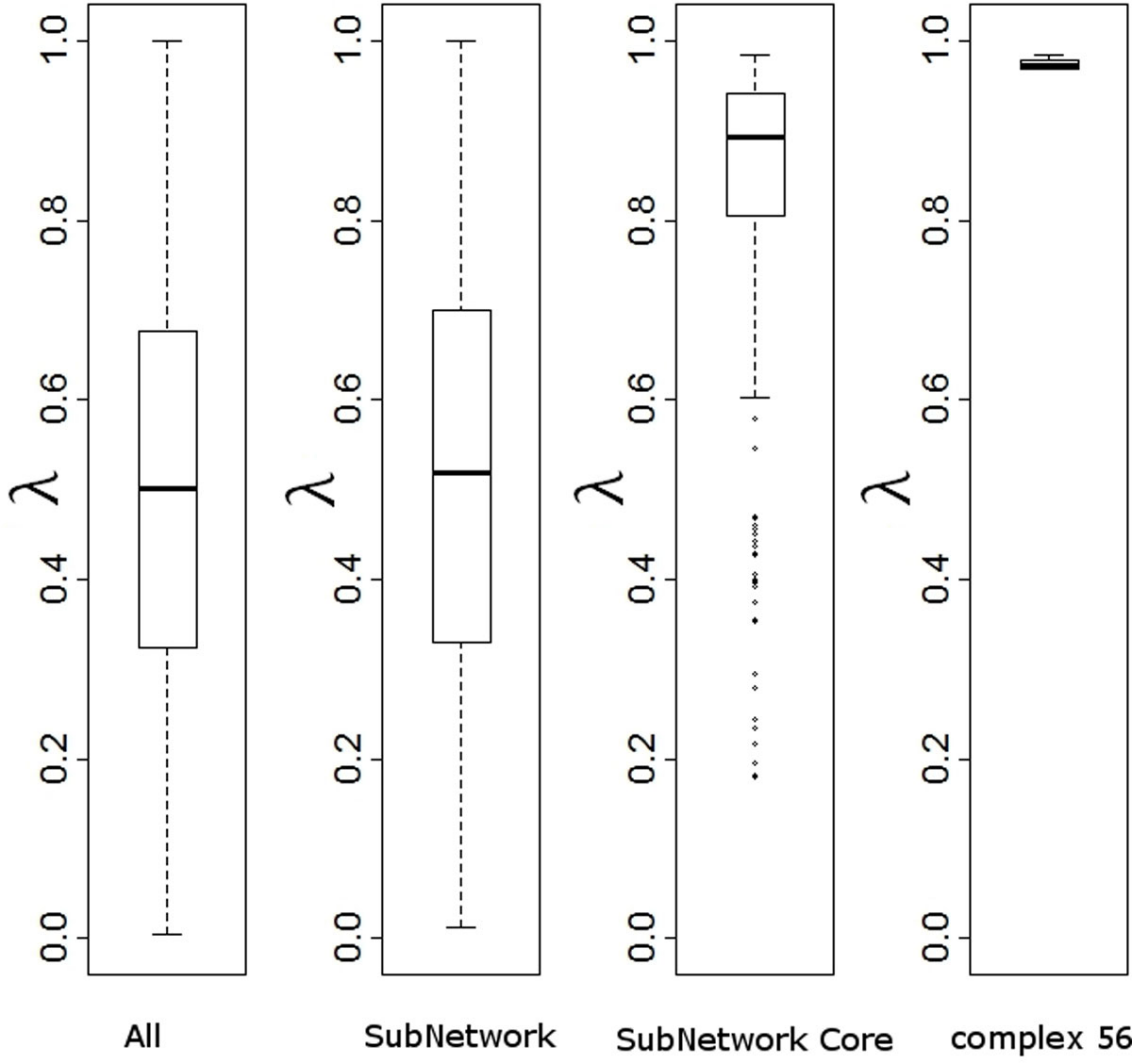
**Boxplot of phase locking index**. Plotted are the mean λ for all interacting gene pairs in PPI; in TopoPL identified subnetwork; in the subnetwork core with the top 30 high degree and high betweenness genes; and in protein complex 56.

We investigated the distribution of the phase locking index within the identified subnetwork. Clearly on average there is a higher degree of phase locking in it than in the whole PPI network (Figure 4). Interestingly the synchronization in the core is even higher, indicating that these core genes may work more closely in a coordinated fashion than others in the identified subnetwork.

### Highly synchronized protein complex

**Figure 5:**
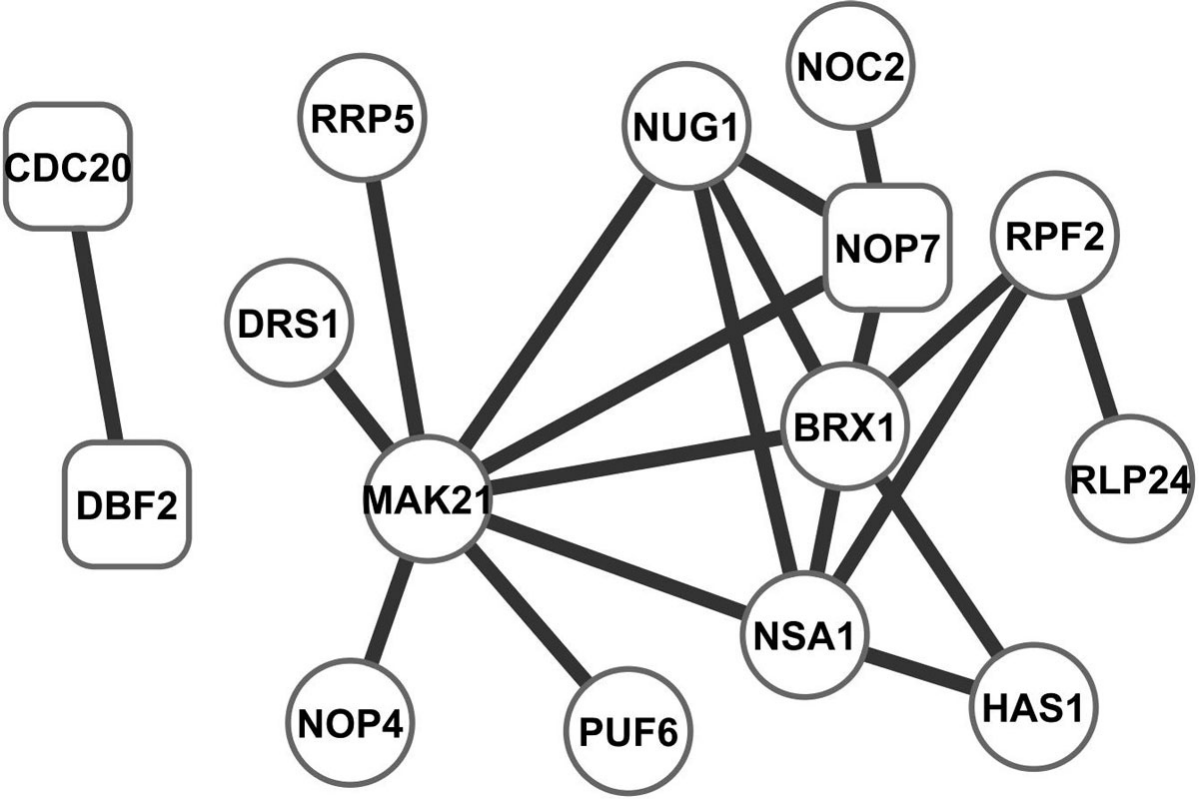
**Top 20 most synchronized Interactions**. Rectangles denote cell cycle genes and thicker lines indicate higher synchronization

We further examined the highly synchronized regions in the network core. Figure 5 shows the top 20 most synchronized interactions (corresponding ~1% of interactions in the identified subnetworks), MAK21 (NOC2) is at the center of this region. MAK21 is involved in preribosome export from the nucleus to the cytoplasm. Though it is not annotated with cell cycle GO term, but its homologue, SWA2 likely plays a role in ribosome biogenesis that is essential for the coordinated mitotic progression [23].

**Figure 6:**
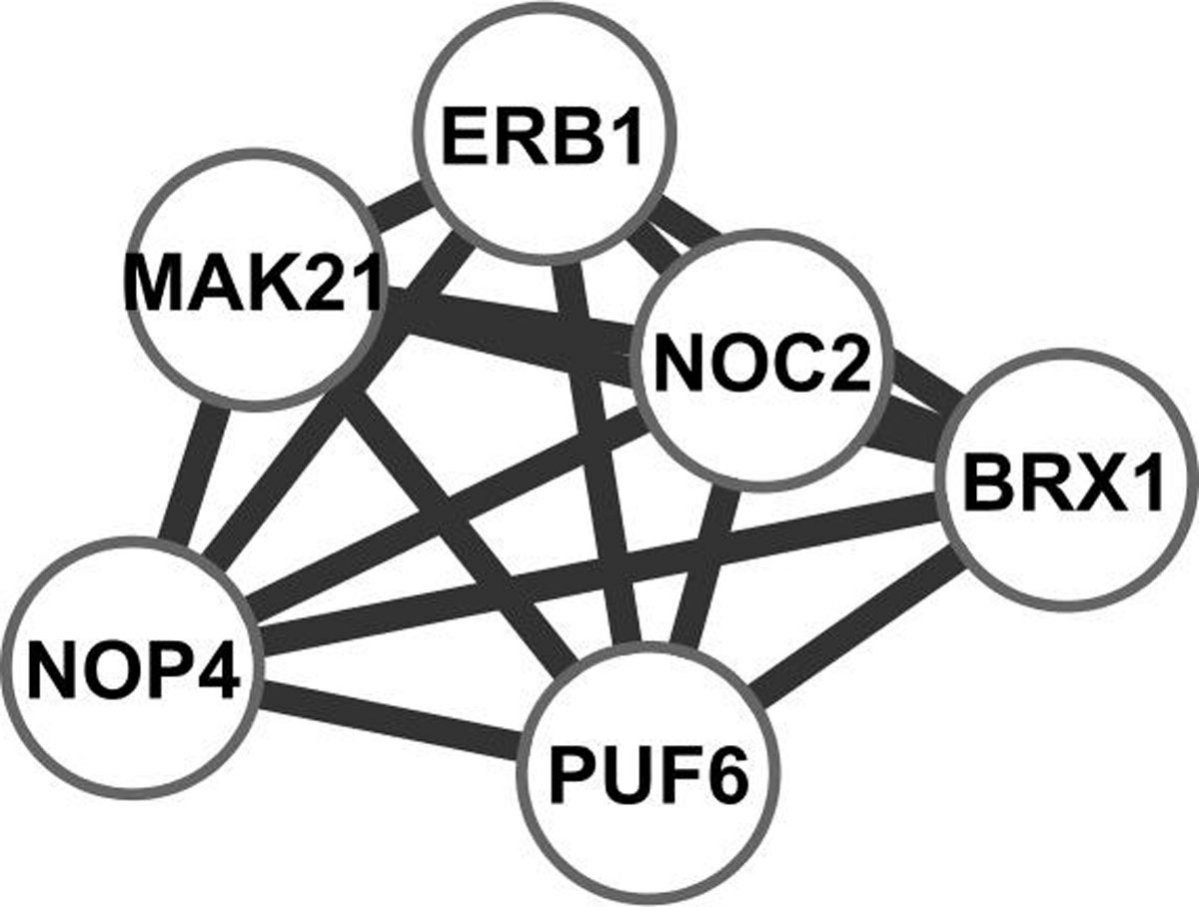
**Interaction network of protein complex 56's core components**.

**Figure 7:**
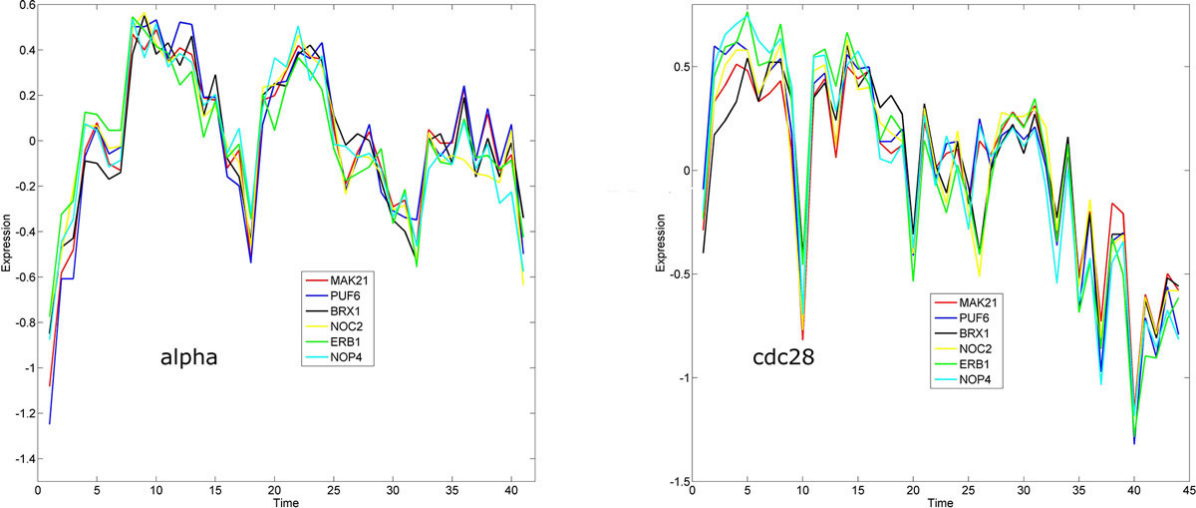
**Expression profiles of genes in the core components of protein complex 56**. Left are the expression profiles in the alpha factor experiment, and right are those in the cdc28 experiment.

In protein complexes, the core components, which consist of two or more proteins that are present in most complex isoforms, are often regarded as functional units as they show surprisingly high degree of functional, essentiality, and localization homogeneity [24,25]. We therefore also surveyed protein complexes and core components in the identified subnetwork. We found that all core components in complex 56 are in our core subnetwork, and they are shown in Figure 6. Interestingly all six genes show extremely high synchronization (0.976±0.006, see Figure 4). Their expression profiles are given in Figure 7. We also included their expression profiles in the cdc28 dataset; again high synchronization in expression is evident. This means that they are coordinated to work closely during cell cycle. This is not surprising as a large percentage of protein pairs within the core subnetwork were coexpressed at the same time during cell cycle [24]. Our algorithm is naturally good at finding highly synchronized genes pairs, therefore tends to include more core components from the same complexes.

Interestingly all six genes are annotated with GO:0042254 (ribosomal chaperone activity), it is defined as "A cellular process that results in the biosynthesis of constituent macromolecules, assembly, and arrangement of constituent parts of ribosome subunits; includes transport to the sites of protein synthesis".

### Transcription factor binding motif analysis

We have found that genes regulated by the same transcriptional factors are likely to be highly synchronized [11]. Here to examine if the reverse is true, we used oPOSSUM (http://opossum.cisreg.ca/oPOSSUM3/) to identify shared transcription factor binding sites (TFBS) among the genes in the identified subnetwork [26]. Given a group of genes, oPOSSUM first detects all TFBS documented in the JASPAR database in promoter regions (1000 bp upstream in this study), and then identifies overrepresented TFBS as compared to background gene sets (all genes in the PPI network in our study). It uses a simple binomial distribution model to compare the rate of occurrence of a TFBS in the set of target genes to the expected rate estimated from the background set. Table 3 gives the top 5 transcription factors of the identified subnetwork and its core.

**Table 3:** Transcription factor binding sites overrepresented in genes of the identified subnetwork and of its core.

The identified subnetwork							Core of the identified subnetwork					
TF	gene hits^1^	gene non-hits	All gene hits	all non-hits	Z-score		TF	gene hits	gene non-hits	All gene hits	all non-hits	Z-score

DOT	131	390^2^	682	4445	38.7		DOT6	6	0	682	4445	25.6

TOD	116	405	639	4488	32.3		TOD6	4	2	639	4488	16.3

FKH	98	423	705	4422	17.0		SFP1	4	2	1203	3924	12.3

SFP1	153	368	1203	3924	15.3		MGA1	3	3	1320	3807	11.1

MCM	346	175	3125	2002	13.7		STB3	4	2	1139	3988	9.14

^1^: "gene hits" is the number of genes that contain the TFBS.

^2^: Note that the sum of columns 2 and 3 is 521, rather than 524, the total number of genes in the subnetwork. This is because that 3 out of the 524 genes do not have entries in oPOSSUM.

FKH1 and MCM1 are well studied cell cycle related transcriptional factors [27]. TOD6 (Pbf1) and DOT6 (Pbf2) as PAC-binding factors, important in the regulation of ribosome biogenesis. Existing ChIP-chip studies suggest that genes have the highest occupancy by TOD6 and DOT6 are highly enriched for the GO Biological Process ''ribosome biogenesis" [28].

### Agreement between the datasets

A good algorithm should be efficient at uncovering the true biology underlying different datasets, which should be consistent. In this study, we identified 484 genes with the cdc28 dataset, and 524 genes with the alpha factor dataset. There are 156 (~31%) overlapping genes in them (p < 0.00001, Fisher Test). In contrast, there are only 87 (~17%) overlapping genes with the Additive method (alpha: 501 genes; cdc28: 509 genes), and 145 (~29%) with TAPPA (alpha: 499 genes; cdc28: 503 genes). This indicates that incorporating network structural and dynamic information can generate robust results.

## Conclusions

TopoPL scoring method with a simulated annealing search was proposed in this study to identify active subnetworks during a biological process by integrating PPI with dynamic expression data. It incorporates both structural and dynamics information of gene interactions. When applied to the simulated data and the yeast cell cycle data, it yielded more consistent results from different experiments, and predicted more meaningful active network modules, than two alternative scoring methods that either ignores information of the network dynamics, or that of both the dynamics and structure.

## Competing interests

The authors declare that they have no competing interests.

## Authors' contributions

SG and XW designed the study. SG wrote the algorithms, performed the analysis, and created the figures and tables. SG and XW wrote the manuscript, read and approved the final version of the manuscript.
